# Evalution of *In Vitro *Effect of Flavonoids on Human Low-Density Lipoprotein Carbamylation

**Published:** 2010

**Authors:** Mohammad Ali Ghaffari, Mehrnoosh Shanaki

**Affiliations:** *Department of Biochemistry, Faculty of Medicine, Ahwaz Jundishapour University of Medical Sciences, Ahwaz, Iran.*

**Keywords:** Low density lipoprotein (LDL), Carbamylation, Flavonoids, Electrophoretic Mobility

## Abstract

The non-enzymatic carbamylation of low density lipoprotein (LDL) is a naturally occurring chemical modification of apolipoprotein B as a result of condensation between lysine residues and cyanate derived from urea. Carbamylated LDL is poorly recognized by LDL receptors and initiates different processes that can be considered proatherogenic. Thus, LDL carbamylation may contribute to the increased risk of atherosclerosis in patients with kidney failure. The objective of this study was to investigate *in vitro *effects of flavonoids on LDL carbamylation. LDL was isolated from plasma using ultracentrifuge technique with a single step discontinuous gradient. Then, cyanate was added to LDL and LDL carbamylation level was estimated in absence and presence of flavonoids by a colorimetric method at 530 nm. The results of this study showed that a number of flavonoids including rutin, catechin, morin, myricetin, kaempferol, taxifolin, luteolin, naringin and quercetin decreased LDL carbamylation in a dose dependent manner. Also, it was demonstrated that these nutrients decreased electrophoretic mobility of carbamylated LDL. Based on the results obtained in this study, it is suggested that flavonoids are able to inhibit LDL carbamylation (probably by scavenging cyanate ions) and thus, may have a role in ameliorating atherosclerotic risk of patients with kidney failure.

## Introduction

 Carbamylation is a spontaneous, non-enzymatic reaction of protein modification by cyanate derived from urea, which is normally present in human plasma and is elevated in uremic patients (1). Urea spontaneously forms cyanate and ammonia, at body pH and temperature (2). Isocyanic acid (the active form of cyanate) reacts irreversibly with the non-protonated amino group of amino acids, forming α-amino-carbamyl-amino acids from free amino acids (3). The irreversible carbamylation forming ε-amino-carbamyl-lysine occurs at multiple lysine sites within a protein with accumulation over the life span of the protein (3). When a molecule of cyanate is removed by carbamylation, a new molecule of cyanate is formed because of the equilibrium between urea and cyanate (3). Chronic kidney disease in humans is associated with a several times increased risk of developing cardiovascular disease because of accelerated atherosclerosis (4). The mechanism of uremia-induced atherosclerosis is not quite understood. It is commonly accepted that endothelial cell injury is an initial event in atherosclerosis (5). Injured endothelial cells attract monocytes, which burrow beneath the endothelial cell layer and ingest modified LDL to form foam cells. This process leads to the formation of atherosclerotic plaque which protrudes into the vessel lumen over the proliferating vascular smooth muscle cells (6, 7). 

Flavonoids are polyphenolic secondary metabolites that are ubiquitous in higher plants (8). Recent epidemiological studies strongly suggest that flavonoid-rich diets are associated with a reduced risk of developing coronary heart disease (9, 10). This potential health benefit has stemmed a lot of investigations on the inhibitory role of flavonoids in the lipid peroxidation of LDL in relation to the implication of oxidized LDL in the formation of the atherosclerotic plaque (11, 12). It has been reported that the human intake of flavonoids from diets is about 1 g/day (13). Flavonoids are of current interest in research due to their important biological and pharmacological properties attributed to their antioxidant properties (14). Nevertheless, the literature data concerning the effect of flavonoids in preventing carbamylate modification of proteins is limited. Given the link mentioned above, we hypothesized that flavonoids might possess anti-carbamylation activities as well. In the present study, we investigated the effect of nine flavonoids (rutin, catechin, morin, myricetin, kaempferol, taxifolin, luteolin, naringin and quercetin) on LDL carbamylation. This study will underline the importance of flavonoids in the prevention of hyperuremia-mediated LDL modification.

## Experimental

**Materials**

rutin, catechin, morin, myricetin, kaempferol, taxifolin, luteolin, naringin and quercetin were purchased from Sigma Chemical Co. (St. Louis, MO, U.S.A). Ethylene diamidine tetraacetic acid (EDTA), dimethyl sulfoxide (DMSO), bovin serum albumin (BSA), agarose, proteinase K, homocitrullin and potassium cyanate (KOCN) were obtained from Merck Chemical Co. (Darmstadt, Germany). Solutions were freshly prepared with double distilled water.

*Preparation of native LDL from plasma*

 Plasma samples were obtained from healthy volunteers after overnight fasting (n = 20, age 25 ± 5 yr, men, non-smoker, non-uremic, not taking any drugs since at least 2 weeks). Native LDL was prepared by ultracentrifugation using a single step discontinuous gradient as described previously (15, 16). The LDL protein content was determined by Bradford method, using bovine serum albumin as standard (17). Cholesterol, triglyceride, LDL-cholesterol and HDL-cholesterol were determined using commercially available Kits (Pars Azmon Kits, Iran). The purity of LDL samples were checked by agarose gel electrophoresis on 0.8% gel (18). Isolated native LDL was kept in darkness under nitrogen gas at 4°C and used within 3 weeks.

*Preparation of carbamylated LDL*

 LDL carbamylation was prepared by in vitro modification of native LDL as described by Weisgraber et al. (19). In short, sterile potassium cyanate was added to the lipoprotein solution at 20 mg/mg of LDL protein. The mixture was incubated at 35°C for 4 h with gentle shaking every hour. Potassium cyanate was removed by extensive dialysis (100-fold volume of dialysis buffer, repeated three times) against 0.15 mol/L, 0.01% EDTA, pH 7.0, for 36 h under sterile conditions at 4°C. Native LDL was dialyzed separately the same way (as control). 

A colorimetric method using diacetyl monoxime was used to measure the degree of carbamylation in LDL preparations (20). Briefly, the LDL suspension (0.6 mg protein/mL) was digested in 160 μL phosphate buffered saline (PBS; 10 mmol/L sodium phosphate buffer pH 7.4, containing 140 mmol/L NaCl) , then 30 μL sodium dodecyl sulfate (10%) and 6 μg proteinase K were added to it and the mixture was incubated at 37°C for 2 h. Then, 800 μL of urea nitrogen reagent (0.83 mol/L sulfuric acid, 1.13 mol/L orthophosphoric acid, 0.55 mmol/L thiosemicarbazide and 2.6 mmol/L cadmium sulfate) and 160 μL diacetyl monoxime (3%) were added to the reaction mixture and incubation continued at 97°C for 30 min. Then, the precipitate was removed by centrifuge at 3500 g for 10 min at room temperature. The absorbance of the supernatant was measured at 530 nm. A standard curve was generated using homocitrullin (ε-amino-carbamyllysine, 0 to 30 nmol). The results were expressed in nmol of homocitrulline per mg of LDL protein. 

*Effect of flavonoids on LDL carbamylation*

The effect of nine flavonoids; rutin, catechin, morin, myricetin, kaempferol, taxifolin, luteolin, naringin and quercetin on the carbamylation of LDL were examined by incubation of LDL (0.6 mg protein/mL) with potassium cyanate (20 μmol/L) and varying concentrations of flavonoids (0-40 μmol/L) in PBS, pH 7.4 at 35°C for 4 hours. The flavonoids were dissolved in 10% DMSO in PBS, at pH 7.4. Degree of LDL carbamylation was determined the colorimetric method as described above (20). The electrophoretic mobility of native and carbamylated LDL and also carbamylated LDL in absence and/or presence of 40 μmol/L of flavonoids were compared by 5% polyacrylamide gel. The gels were stained with Coomassie blue stain (18). 

*Statistical analysis*

Results are expressed as means ± SD. Carbamylated modification of LDL in the absence (as control) or presence of flavonoids was compared using analysis of variance (ANOVA). A value of P<0.05 was considered significant. 

## Results

**Figure 1:**
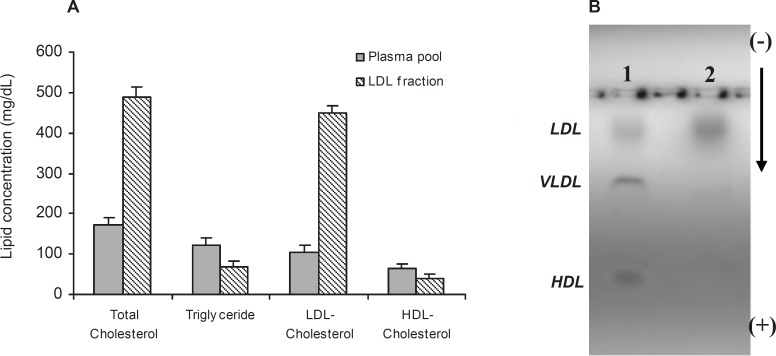
(A) †Comparison of lipid concentrations in plasma pool and LDL fraction. (B) Electrophoresis of plasma (Lane 1) and LDL fraction (Lane 2) on 0.8% agarose gel. *Values have represented as mean ± SD of triplicate determinations. LDL=Low density lipoprotein,* *VLDL=Very low density lipoprotein, HDL=High density lipoprotein*

Isolation of LDL from plasma was confirmed by measurement of lipid concentration and agarose gel electrophoresis (Figure 1). As shown in Figure 1A cholesterol and LDL cholesterol amounts were increased in LDL preparation approximately by 65% and 77%, respectively. 

**Figure 2:**
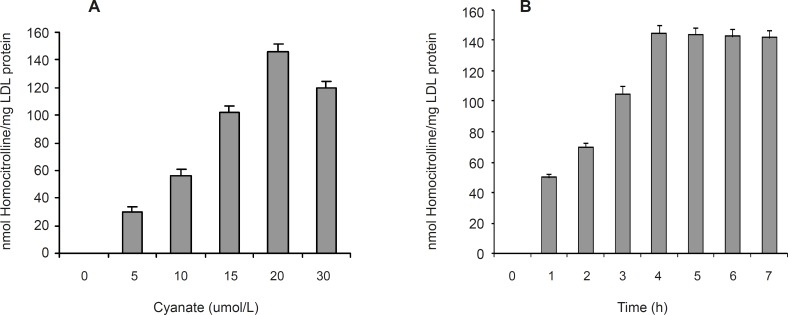
The effect of cyanate concentration (A) and incubation time (B) on carbamylation of LDL. Values have represented as the means ± SD of triplicate determinations

Figure 1B also shows the separated fractions of LDL (Lane 2) compared to plasma (Lane 1). Carbamylated LDL was prepared by incubation of LDL with potassium cyanate as in vitro. In this study, the optimum concentration of potassium cyanate for carbamylation of LDL was investigated by incubation of a range of cyanate concentration (0-30 μmol/L) with LDL (0.6 mg protein/mL) in PBS (10 mmol/L, pH 7.4) at 35°C for 4 h (Figure 2A). The best incubation time for LDL carbamylation was also investigated by incubation 0.6 mg protein/mL of LDL with 20 μmol/L cyanate for 1 to 7 h at 35°C (Figure 2B). As shown in Figure 2, optimum cyanate concentration and incubation time were obtained for LDL carbamylation 20 μmol/L and 4 h, respectively.

**Figure 3:**
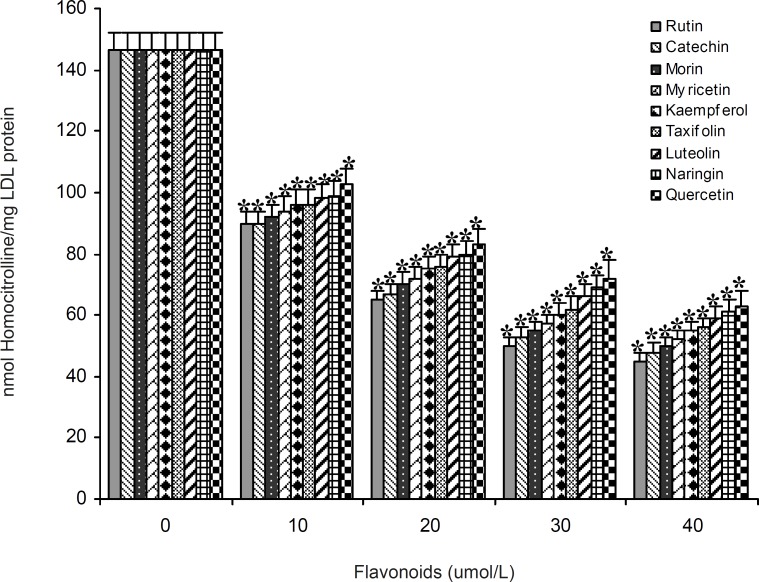
The effect of 10 to 40 μmol/L concentrations of 9 flavonoids (rutin, catechin, morin, myricetin, kaempferol, taxifolin, luteolin, naringin and quercetin) on carbamylation of LDL (0.6 mg protein/mL) by cyanate (20 μmol/L). Values have represented as the mean±SD of triplicate determinations. ^*^P<0.001 compared with control (in absence of flavonoids

**Figure 4:**
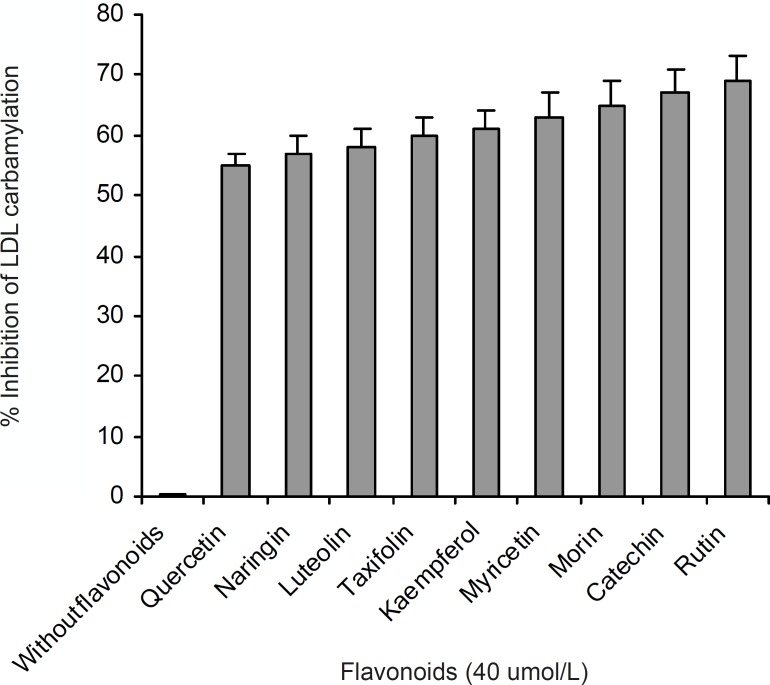
Comparison of the percent inhibition of LDL carbamylation in absence and presence of 40 μmol/L concentration of flavonoids. This figure was obtained by Figure 3 data

**Figure 5:**
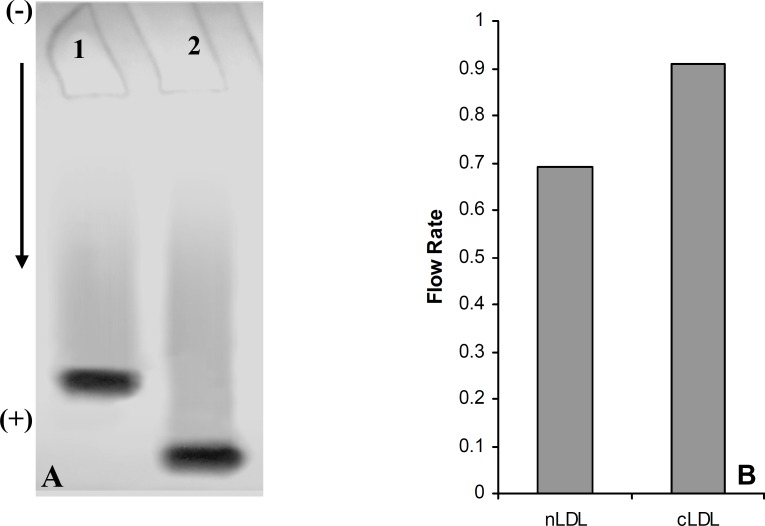
(A) Electrophoresis analysis of native LDL (Lane 1) and carbamylated LDL (Lane 2) on 5% polyacrylamide gel. (B) Comparison of the flow rate of native LDL (nLDL) with carbamylated LDL (cLDL) on 5% polyacrylamide gel

**Figure 6:**
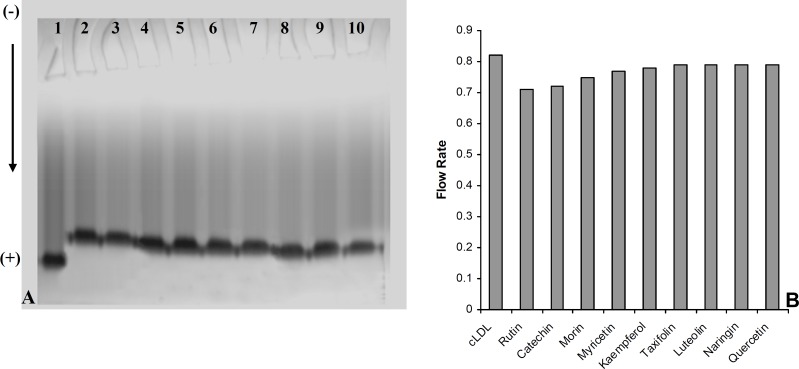
(A) Electrophoresis analysis of carbamylated LDL in absence (Lane 1) and presence of 40 μmol/L concentration of rutin (Lane 2), catechin (Lane 3), morin (Lane 4), myricetin (Lane 5), kaempferol (Lane 6), taxifolin (lane 7), luteolin (Lane 8), naringin (Lane 9) and quercetin (Lane 10) on 5% polyacrylamide gel. (B) The Comparison of the flow rate of carbamylated LDL (cLDL) in absence and presence of flavonoids (40 μmol/L) on 5% polyacrylamide gel

A series of experiments were performed to examine the influence of flavonoidson LDL carbamylation process. Rutin, one of the flavonoids tested, was incubated at concentration of 0 to 40 μmol/L with LDL (0.6 mg protein/mL) and cyanate (20 μmol/L) at 35°C for 4 h. The extent of LDL carbamylation in the absence (as control) and/or presence of rutin were estimated as nmol homocitrolline per mg LDL protein (Figure 3). The same procedure was repeated to investigate the effect of other flavonoids. Figure 3 shows the inhibitory effect of 9 flavonoids (5 flavonols (kaempferol, morin, rutin, myricetin, quercetin), 1 flavonone (naringin),, 1 flavone (luteolin), 1 flavanol (catechin) and 1 flavanolol (taxifolin)) on LDL carbamylation. The results presented in Figure 3 showed that these flavonoids, compared to the controls, decrease significantly the LDL carbamylation in a dose-dependent manner. In this study, all flavonoids in comparison to the control (without flavonoids) were shown a significant inhibition of LDL carbamylation as shown by the ANOVA test, P < 0.001. According to these study, 40 μmol/L concentrations of rutin, catechin, morin, myricetin, kaempferol, taxifolin, luteolin, naringin and quercetin are able to reduce LDL carbamylation approximately by 69%, 67%, 65%, 63%, 61%, 60%, 58%, 57% and 55%, respectively (Figure 4). We also investigated electrophoretic mobility of 20 μmol/L cyanate treated LDL on polyacrylamide gel (Figure 5). Figure 5 shows that carbamylation increased anodic migration (flow rate) of LDL when compared to native LDL. The comparison of electrophoretic mobility of carbamylated LDL in presence of flavonoids (40 μmol/L) showed a decrease in anodic migration and/or flow rate of this LDL isoform (Figure 6). These observations suggest that flavonoids above can decrease LDL carbamylation in presence of cyanate.

## Discussion

Urea is a normal component of human blood plasma where it undergoes spontaneous transformation to cyanate. With the decrease of renal function, there is an increased amount of urea and cyanate acts as a uremic toxin through the carbamylation of proteins (1). Cyanate can react irreversibly with the free ε-amino groups of lysine and N-terminal amino acids within proteins. The resulting in vivo carbamylation can change the structure and activity of proteins (21). Weisgraber et al. described carbamylated LDL interact with cell surface receptors in human fibroblasts and prevent the binding of native LDL in human fibroblasts (19). Horkko et al. showed that LDL isolated from uremic patients as well as carbamylated LDL had a slower clearance than normal subjects or non-modified LDL (22). 

Although carbamylation of LDL has already been found in uremic patients (7, 24), the effects of flavonoids on LDL carbamylation have not been previously studied. Roberts and Harding discussed that ibuprofen protects lens against cataract by reduction of cyanate binding to lens proteins (25). Similar study showed by Crompton et al. who discussed that aspirin decreases the phase separation temperature in lenses exposed to cyanate and was found to reduce rate of carbamylation of most soluble lens proteins (26). Several reports suggest that uremia is a state of oxidative stress. These include findings such as an increase in lipid peroxidase in uremic red blood cell membranes and a decrease in serum antioxidant activity (27, 28). Thus, we hypothesized that antioxidant flavonoids might possess anti-carbamylation activity.

In this study, the phenomenon of LDL carbamylation was demonstrated in the reaction mixtures of LDL with cyanate by in vitro model system*. *Apo-B_100 _of LDL, the amine source could serve as target for carbamylated agents. Our experiments were performed with various concentrations of cyanate (0-30 μmol/L) on LDL carbamylation. The carbamylation of LDL was increased in the presence of 5 to 20 μmol/L of cyanate concentrations, and was decreased, however, at higher concenetration of cyanate (30 μmol/L). Our results showed that cyanate at concentration of 20 μmol/L has maximum effect on carbamylation of 0.6 mg the protein per mL of LDL concentration. These findings suggest novel treatments for the prevention of atherosclerosis in uremic patients, which are based on the prevention of LDL carbamylation and/or reduce the effects of carbamylation. Thus, in order to evaluate the effects flavonoids on LDL carbamylation by cyanate, we conducted the presnt investigation. Flavonoids were chosen in this study as they are rather universally found in herbal foods (8, 23). Nine flavonoids of different chemical structures were investigated in this study, including flavonols (kaempferol, morin, rutin, myricetin and quercetin), flavone (luteolin), flavanol (catechin), flavonone (naringin) and flavanolol (taxifolin). Our results revealed that the nine flavonoids with concentrations of 10 to 40 μmol/L significantly reduced susceptibility of LDL to carbamylation (n = 3, ANOVA test, P < 0.001). A marked reduction of carbamylation was observed with rutin (40 μmol/L) treatment. 

The suppression of LDL carbamylation by nine flavonoids, expressed as percentage, were as follow: rutin(69%) > catechin(67%) > morin (65%) > myricetin(63%) > kaempferol(61%) > taxifolin(60%) > luteolin(58%) > naringin(57%)> quercetin (55%). It was found that when LDL was subjected to cyanate-mediated modification, the addition of flavonoids decrease nmol level of homocitrulline released per mg of LDL protein, and this effect was concentration dependent. Rutin, with rhamnose-glucose group at C-3 position in the C ring and with hydroxyl groups at the C-5, 7 and 3’, 4’ positions in the B ring (29) was the most effective compound in our study. Other eight flavonoids lack the C-3 glucosyl group, thus it may indicate that glucoside group at C-3 position in C ring would be the necessary functional group for inhibition of carbamylation. Our study also showed that the mobility of carbamylated LDL increased on polyacrylamide gel, which probably resulted from the increased negative charge caused by the modification of lysine amino groups. The increased electrophoretic mobility is in agreement with the finding reported by Witztum et al. (30). The presence of flavonoids decreased the flow rate of carbamylated LDL on 5% polyacrylamide gel, approximately followed the order of rutin (13.5%) > catechin(12.2%) > morin (8.5%) > myricetin (6%) > kaempferol (4.9%) > taxifolin (3.6%) ≥ luteolin (3.6%) ≥ naringin (3.6%) ≥ quercetin (3.6%). Thus, these results support the suggestion that the nine flavonoids mentioned above play an important role in prevention of LDL carbamylation by cyanate. The mechanism by which flavonoids suppress carbamylation of LDL is still not known. Smyth indicated that hydroxyl group of tyrosine as a nucleophilic factor is able to bind to cyanate (31). Thus, we could suggest that anti-carbamylation activity of flavonoids is possibly related to their abilities to scavenge cyanate.

In conclusion, the results obtained in the present study showed that all nine flavonoids of interest, especially rutin, have inhibitory effects on LDL carbamylation. The scavenging of cyanate, derived from urea, may play an important role in this phenomenon. Therefore, flavonoids which prevent LDL carbamylation and/or reduce the effects of carbamylation, may provide novel treatments for the prevention of atherosclerosis in uremic patients. 
